# Towards a European “Cohort Moonshot”: revisiting the long-term strategy to support health research of tomorrow

**DOI:** 10.1007/s10654-022-00939-5

**Published:** 2023-01-04

**Authors:** Guy Fagherazzi

**Affiliations:** Deep Digital Phenotyping Research Unit, Department of Precision Health, Luxembourg Institute of Health, 1 A-B Rue Thomas Edison, 1445 Strassen, Luxembourg

**Keywords:** Epidemiology, Cohort, Precision Health, Health Data, Digital Health, Public Health

At a time when global access to healthcare is not secured and grave health inequalities persist, the implementation of evidence-based personalized health strategies remains nothing but a utopia. The promised revolution of artificial intelligence (AI) and digital health for personalized healthcare has yet to happen. We are even far from the adoption of machine learning approaches to stratify the patients and populations to define personalized prevention strategies. One of the main causes of this impasse is access to high-quality and standardized data. AI algorithms are often trained on low-quality, biased datasets with limited sample sizes, and the resulting models fail to generalize to the larger population and reach clinical maturity. Facilitated access to large and thoroughly characterized cohorts would, at least partially, solve these issues.

Today’s major advancements in epidemiology and clinical research originate from large population or patient-based cohort studies. Countries who had the vision of implementing mega cohorts early, such as the UK Biobank (UK, 500,000 participants) [1], the All of Us Research Program (USA, up to 1 million participants), Constances (France, 200,000 participants) [2], or the German National Cohort NAKO (Germany, 205,000 participants) [3] are now collecting the fruits of their massive investments with breakthrough scientific achievements.

Regarding most cohort studies, except maybe the example of the UK Biobank, there is a cohort paradox: large volumes of data from cohorts already exist in research centers or hospitals but are largely underexploited. The data sometimes remain behind obscure data and sample access procedures, limiting the reach and accessibility of such resources by external scientists. But this is easily understandable: teams and PIs who have shed blood and tears to create and maintain these research infrastructures for many years are not compensated enough for their accomplishments. They feel frustrated to see external researchers exploiting the data and being acclaimed for their publications once all the heavy lifting is done.

Cohorts, by design, are tools that have a relatively long time of return on investment [4]. The E3N cohort, initiated in 1990 in France, is one of the most extensive cohort studies on women’s health and is still active with 32 years of follow-up [5]. During the first five years of the cohort, virtually no peer-reviewed publication came out. The scientific community frequently mocked the project, when case–control studies were the most frequent study design. Today, this large research infrastructure enabled the publication of more than 1100 publications including major findings in cancer and chronic disease prevention as well as in pharmaco-epidemiology.

With the current international organization, where cohort studies are run in silos, there is an important waste of public money and as such, an important opportunity in terms of economy of scale. This is particularly visible when it comes to patient-based cohorts where everyone is trying to get their “own” cohort, collect data and samples and try to secure funds for a few research projects, after which the follow-up is often stopped due to lack of resources or staff to maintain them. Collectively, this represents a major waste of resources, and can even be considered as an unethical approach with respect to the time and data that the participants have shared with a hope to significantly contribute to research.

We need a “cohort moonshot” program to create a sustainable model to support the next generations of cohort studies. European grants or large national funding schemes are often used to initiate cohort studies, but, because they are restricted in time, they are not appropriate to support cohorts in the long run.

As such, we should move from a sporadic and fragmented funding mechanism to a continuous support principle for data and sample generation in the long run. The European Prospective Investigation into Cancer and Nutrition (EPIC) study is a perfect example. Started in 1990 by the IARC and WHO, EPIC is still one of the largest cohort studies in the world today, with more than half a million participants recruited across 10 European countries [6]. Initially funded by the European Commission and national sources, the follow-up of the participants stopped in 2015. The cohort is still used by the EPIC consortium today (more than 1800 peer-reviewed publications indexed in Pubmed used EPIC data), but its value will inevitably decrease over time without a substantial, sustainable funding mechanism to update the cohort and expand the data and sample collection. What will likely become a waste of invaluable research data and samples in the mid-term could be turned, with some vision, into the basis of a long term, European Health cohort.

Changing the economic model of mega cohorts and the compensation mechanisms for the teams in charge of cohort implementation is a necessity. Despite the slow upcoming paradigm shift in scientific evaluation (moving away from H-index and number of publications towards a better societal impact), collecting data and generating research resources accessible to other researchers is not sufficiently recognized in the research community. How can you conciliate the promotion of Open Science and Open Data practices if, on the other hand, the producers of data are not financially compensated for their efforts and not incentivized to maintain and develop their cohort studies?

If we consider cohorts as long term tools to improve public health, we could argue that creating large, high quality and sustainable research infrastructures should be a task delegated to a governmental, national or European entity, and no longer driven by a group of academic researchers.

To fully embrace our present and future societal and healthcare challenges, we should start funding a “Cohort Moonshot” and implementing a European Health Cohort, with a “One Health” approach, invest in digital and IT technologies to facilitate the data capture and ensure the trust of the citizens, and rely on the European Health Data Space. The collection of data and samples in a federated and privacy preserving fashion is technically possible and it would allow the follow-up of millions of citizens' health parameters for research purposes. The anticipated era of precision health might then become a reality.
